# The role of the microbiome in ovarian cancer: mechanistic insights into oncobiosis and to bacterial metabolite signaling

**DOI:** 10.1186/s10020-021-00295-2

**Published:** 2021-04-01

**Authors:** Adrienn Sipos, Gyula Ujlaki, Edit Mikó, Eszter Maka, Judit Szabó, Karen Uray, Zoárd Krasznai, Péter Bai

**Affiliations:** 1 Department of Medical Chemistry, Faculty of Medicine, University of Debrecen, Debrecen, 4032 Hungary; 2 Department of Gynecology and Obstetrics, Faculty of Medicine, University of Debrecen, Egyetem tér 1, Debrecen, 4032 Hungary; 3 Department of Medical Microbiology, Faculty of Medicine, University of Debrecen, Debrecen, 4032 Hungary; 4 MTA-DE Lendület Laboratory of Cellular Metabolism, Debrecen, 4032 Hungary; 5 Research Center for Molecular Medicine, Faculty of Medicine, University of Debrecen, Debrecen, 4032 Hungary

**Keywords:** Ovarian cancer, Microbiome, EMT, Microbial metabolite, Indole derivative, Lipopolysaccharide, Lysophosphatid, Antibiotic, Probiotic

## Abstract

Ovarian cancer is characterized by dysbiosis, referred to as oncobiosis in neoplastic diseases. In ovarian cancer, oncobiosis was identified in numerous compartments, including the tumor tissue itself, the upper and lower female genital tract, serum, peritoneum, and the intestines. Colonization was linked to Gram-negative bacteria with high inflammatory potential. Local inflammation probably participates in the initiation and continuation of carcinogenesis. Furthermore, local bacterial colonies in the peritoneum may facilitate metastasis formation in ovarian cancer. Vaginal infections (e.g. *Neisseria gonorrhoeae* or *Chlamydia trachomatis*) increase the risk of developing ovarian cancer. Bacterial metabolites, produced by the healthy eubiome or the oncobiome, may exert autocrine, paracrine, and hormone-like effects, as was evidenced in breast cancer or pancreas adenocarcinoma. We discuss the possible involvement of lipopolysaccharides, lysophosphatides and tryptophan metabolites, as well as, short-chain fatty acids, secondary bile acids and polyamines in the carcinogenesis of ovarian cancer. We discuss the applicability of nutrients, antibiotics, and probiotics to harness the microbiome and support ovarian cancer therapy. The oncobiome and the most likely bacterial metabolites play vital roles in mediating the effectiveness of chemotherapy. Finally, we discuss the potential of oncobiotic changes as biomarkers for the diagnosis of ovarian cancer and microbial metabolites as possible adjuvant agents in therapy.

## Background

Ovarian cancer is leading oncological cause of death among women. Ovarian cancer is characterized by changes to different microbiome compartments that is termed oncobiosis. The aim of the current work is to provide a comprehensive review of changes to microbiome and to give mechanistic insights to the role of the microbiome in the pathogenesis of ovarian cancer. These mechanistic steps involve, but are not limited to the induction of sustained inflammation and the production of procarcinogenic bacterial metabolites. The interference between the oncobiome and the chemotherapeutic agents will be discussed, as well as, the possible application of antibiotics, probiotics and nutrients in the management of ovarian cancer.

## Etiology and treatment of ovarian cancer

Ovarian cancer is the second most common gynecological malignancy in developed countries and has one of the worst prognosis and mortality (Torre et al. 2015). Most ovarian tumors, approximately 90%, are of epithelial origin (Heintz et al. 2001; Banks 2001). According to the presently accepted dualistic model, ovarian cancer is not a single homogenous disease, but consists of two major types, type I and II (Kurman 2013). Type I tumors (30%) are low-grade tumors, which are clinically slow-growing large cystic formations with mutations in KRAS, BRAF, PTEN, CTNNB1, PIK3CA, PPP2R1A, and ARID1A genes (Shih Ie and Kurman 2004; Wiegand et al. 2010). On the contrary, type II tumors, accounting for 70% of all ovarian malignancies, are aggressive high-grade cancers that almost always present as advanced stage with high fatality. Type II tumors have alterations in the TP53, BRCA1, BRCA2 genes and have very high genetic instability (Kurman 2013; Kurman and Shih 2011). Approximately 90% of type II tumors are high-grade serous cancers (HGSC) and are derived from serous tubal interstitial carcinoma (STIC) of the fallopian tube (Kurman 2013; Carlson et al. 2008; Kindelberger et al. 2007). Hereditary factors are responsible for about 20% of all ovarian cancers, stemming from mostly BRCA1 and BRCA2 mutations (Lynch et al. 1993; Risch et al. 2006; Medeiros et al. 2006). Somatic alterations of genes in the homologous repair pathways are more frequent than those of germ-line origin. More than 2/3 of patients present with advanced-stage (FIGO III-IV) disease.

A complete tumor reduction, called primary debulking surgery, is the cornerstone of initial treatment for ovarian cancer. Tumor reduction may necessitate multiple organ resections (bowel resection, peritonectomy, and splenectomy) (Chang et al. 2012; Querleu et al. 2017). The goal is to achieve “optimal” cytoreduction, which is defined as “no residual macroscopic disease”, according to the Vancouver Consensus Conference 2010 (Stuart et al. 2011). If optimal tumor reduction is not feasible, neoadjuvant intravenous chemotherapy with paclitaxel and carboplatin is initiated and an interval debulking surgery (IDS) is performed, if a partial or complete response is observed after 3 cycles (Querleu et al. 2017; Vergote et al.^2010^; Zeng et al.^2016^; Fagotti et al. 2016). Since neoadjuvant chemotherapy increases the rate of complete tumor reduction, but does not improve survival, it is only non-inferior to upfront surgery, which is preferred if possible (Morrison et al. 2012; Medina-Franco et al. 2017). In cases of HGSC, surgery is followed by adjuvant chemotherapy.

The standard of care for the past 20 years is a combination of paclitaxel and platinum, which is routinely administered intravenously (Jayson et al. 2014). This therapy is superior, as the first-line treatment of ovarian cancer, over any other drug combination (Kyrgiou et al. 2006). The modified dose-dense treatment with weekly paclitaxel regimen further improves survival, although side effects are more severe (Katsumata et al. 2009). Although intraperitoneal chemotherapy seems to have survival benefits over intravenous administration according to some trials, intraperitoneal treatment has a higher complication rate (e.g. catheter-related problems) and is not routinely used, although the option is open for select cases (Jaaback and Johnson 2006).

Angiogenesis plays a very important role in the peritoneal spread and metastasis forming potential of ovarian cancer (Yoneda et al. 1998). Therefore, targeted therapies against vascular endothelial growth factor (VEGF) have important therapeutic effects (Burger et al. 2011). If tumor reduction is not complete during surgery, patients receive bevacizumab, an anti-VEGF monoclonal antibody (NCCN Guidelines). Bevacizumab prolongs progression-free survival and quality of life. However, bevacizumab is beneficial for overall survival only in poor prognosis groups (Stark et al. 2013; Tewari et al. 2019).

Poly[ADP-ribose] polymerase (PARP) plays an essential role in DNA repair (Curtin and Szabo 2013). Patients with germline or somatic BRCA1/2 mutations who show partial or complete response to platinum chemotherapy receive PARP inhibitors (olaparib or niraparib) as a maintenance therapy (NCCN Guidelines) [for an overview on the current studies see (Curtin et al. 2020; Mateo et al. 2019; Curtin and Szabo 2020)]. Even patients without known BRCA 1/2 mutations may benefit from maintenance niraparib therapy after first-line treatment, because other homologous repair defects may be present in the tumor (NCCN Guidelines). If bevacizumab is part of the primary therapy, the addition of olaparib in combination with maintenance therapy gives a significant progression-free survival benefit to patients regardless of BRCA1/2 mutation status (Ray-Coquard et al. 2019).

Despite initial therapy, the disease recurs in about 70–80% of advanced-stage patients, and the 10 year disease-free survival rate is below 15% in these patients (Coleman et al. 2019; Dood et al. 2018). If the disease recurs 12 months or later after the end of platinum therapy, the tumor is “platinum sensitive”, in a range between 6 and 12 months the tumor is “partially platinum sensitive”, and recurrence within 6 months means “platinum resistant” disease. Patients with platinum sensitive recurrence receive platinum reinduction therapy with paclitaxel and carboplatin, while the management of platinum-resistant disease is a much greater challenge. In the latter cases, single agent paclitaxel, topotecan, and pegylated liposomal doxorubicin (PLD) remain an option (Bergamini et al. 2019). These drugs can also be given in combination with bevacizumab as second-line therapy (Poveda et al. 2015). In addition, PARP inhibitors have an important role in the management of recurrent disease (NCCN Guidelines; Ledermann et al. 2012; Mirza et al. 2016). There is strong evidence that secondary cytoreductive surgery does not improve survival of recurrent ovarian cancer patients (Coleman et al. 2019).

## Interactions between the oncobiome and cancer

A large set of neoplastic diseases are characterized by changes to microbiome compartment(s) that is termed oncobiosis, the opposite of which is eubiosis. Oncobiosis has a role in the pathogenesis of neoplastic diseases. Microbiome-neoplastic cell interactions are multi-pronged (Miko et al. 2016; Miko et al. 2019; Zitvogel et al. 2017; Kovacs et al. 2020; Finlay et al. 2020) and can impact on multiple cancer hallmarks (for cancer hallmarks, see the seminal papers of Hanahan and Weinberg (Hanahan and Weinberg 2011, 2000)). Microbiome-related effects stem from basic cellular functions, such as changes to redox homeostasis (Kovács et al. 2019; Smolková et al. 2020; Sári et al. 2020a, b) or changes to cellular metabolism (Sári et al. 2020a, b; Miko et al. 2018; Kovács et al. 2019), via altered gene expression patterns. These primary changes then modulate larger scale events, namely, epithelial-to-mesenchymal transition (Sári et al. 2020b; Miko et al. 2018; Kovács et al. 2019; Buchta Rosean et al. 2019; Ingman 2019; Vergara et al. 2019), cancer cellular movement, invasion, diapedesis and metastasis formation (Kovács et al. 2019; Sári et al. 2020a, b, 2020; Miko et al. 2018; Kovács et al. 2019), angiogenesis (Miko et al. 2018), the modulation of antitumor immunity (Sári et al. 2020a; Miko et al. 2018; Vergara et al. 2019; Sipe et al. 2020; Osman and Luke 2019; Zitvogel et al. 2016; Routy et al. 2018a, 2018b; Gopalakrishnan et al. 2018; Elkrief et al. 2019; Derosa et al. 2020; Hall and Versalovic 2018; Viaud et al. 2014), and tumor-promoting inflammation (Kiss et al. 2020; Yu 2018).

The elementary events act together and the result of their action is dependent on the circumstances. A good example is oxidative stress or the modulation of the immune system. Sustained oxidative stress induces DNA damage and the accumulation of mutations increases the risk for carcinogenic transformation (Smolková et al. 2020; Lau et al. 2008; Jezierska-Drutel et al. 2013). In this case, the dysbiotic microbiome drives local inflammation upon pathological colonization, such as in ovarian carcinoma (Wang et al. 2020) or pancreas adenocarcinoma (Kiss et al. 2020). On the contrary, low oxidative stress, induced by bacterial metabolites, can exert cytostatic (but not cytotoxic) properties, as in the downregulation of NRF2 in breast cancer (Kovács et al. 2019; Sári et al. 2020a, b).

Similar to the aforementioned oxidative stress, the immune system is a double-edged sword. The oncobiome has different effects on the immune system than the eubiome. Bacteria themselves can act as baits for the immune system. Furthermore, immunomodulatory bacterial metabolites were identified in multiple carcinomas (Sári et al. 2020a; Miko et al. 2018) that can fine tune the behavior of the immune system. Hence, the oncobiotic transformation may tune the immune system differently (Zitvogel et al. 2016). The tolerogenic state of the immune system jeopardizes the early elimination of cancer cells, reduces the efficiency of immunotherapy, and reduces oxidative stress (Zitvogel et al. 2016). In contrast, a more immunogenic microbiome supports immunotherapy (Routy et al. 2018a; Gopalakrishnan 2018), but in turn induce higher oxidative stress and increase the risk for mutations and may sustain tumorigenic inflammation (Buchta Rosean et al. 2019; Pagliari et al. 2018; Ochi et al. 2012; Pushalkar et al. 2018; Sethiet al. 2018; Ren et al. 2017).

What can drive oncobiotic transformation? Lifestyle choices are major contributing factors, including smoking (Biedermann 2013), feeding, obesity (Schulz et al. 2014), changes to the diurnal rhythm (Zarrinpar et al. 2016, 2014; Paschos and FitzGerald 2017), aging (Zhang et al. 2019; Saffrey et al. 2014; Singh et al. 2019), underlying diseases such as diabetes (Devaraj et al. 2013), and exercise (Ticinesi et al. 2019). In addition, antibiotic (Friedman et al. 2006) or probiotic use (Mendoza 2019; Ranjbar et al. 2019) are associated with carcinogenesis. Recently, interbacterial signaling was identified, which depends on the release of components of bacterial cells that trigger resistance of the remaining live cells to the noxa that causes bacterial cell death (Bhattacharyya et al. 2020). The involvement of “dead cell signaling” has not been evaluated in controlling the composition of the microbiome. Sensing the numbers of bacteria in the environment (quorum sensing) is also a major player in fine tuning the microbiome (Li et al. 2019; Juhász et al. 2017).The microbiome also interferes with all therapeutic modalities, including chemotherapy, radiotherapy, and targeted therapeutic approaches (Bashiardes et al. 2017; Alam et al. 2020; Roy and Trinchieri 2017). Interestingly, while bacteria can interfere with the metabolism or distribution of the elements of therapy (Perales-et al. Puchalt 2018), therapy can modulate the composition of the microbiome.

## Oncobiotic transformation in ovarian cancer

**Fig. 1:**
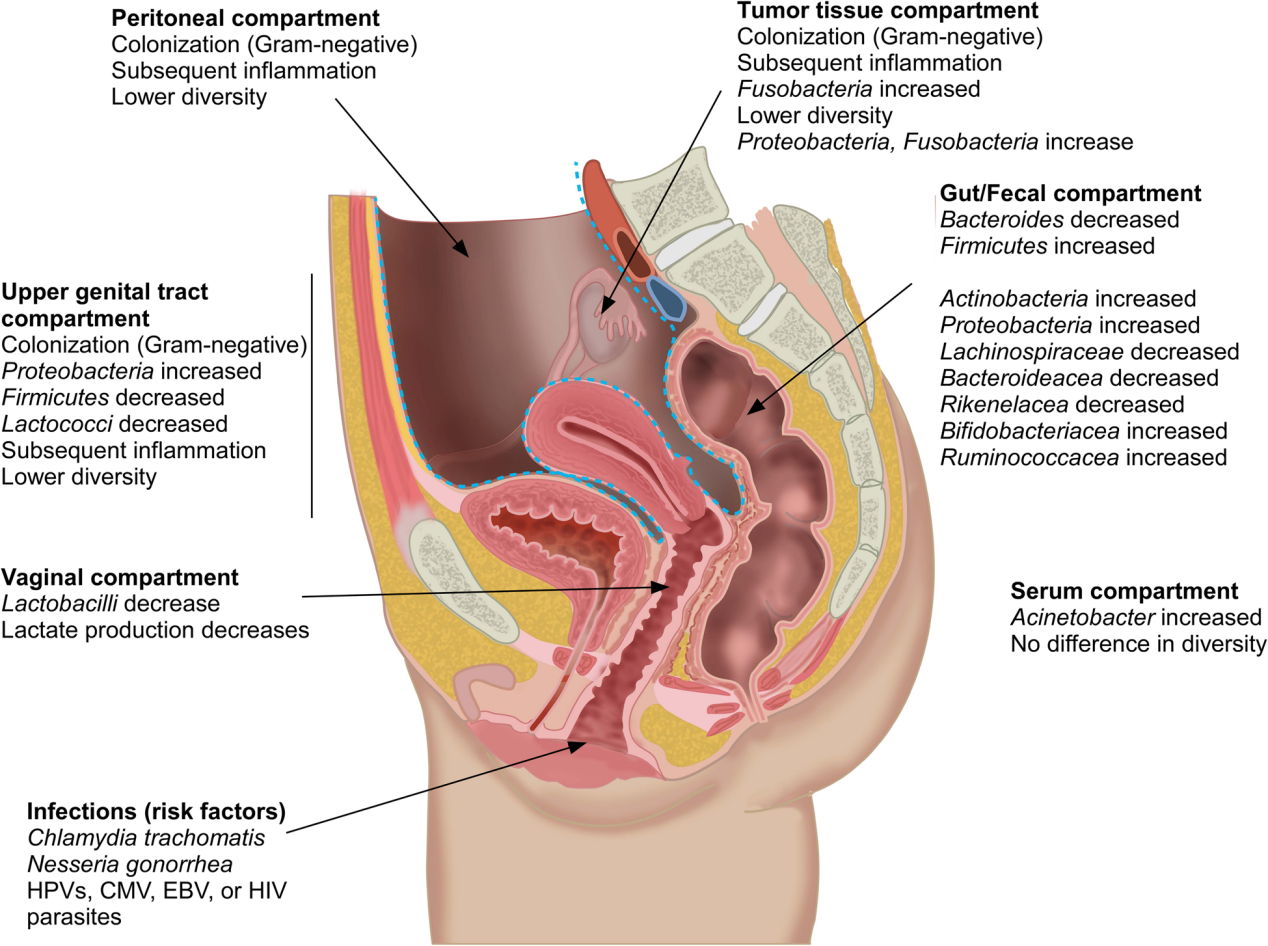
Changes to microbiome compartments in ovarian cancer. The center figure is taken from https://anatomytool.org/content/sagittal-section-female-pelvis-peritoneum as a free image

Oncobiosis was identified in several compartments, including vaginal, cervicovaginal (Ness et al. 2003; Nené et al. 2019), upper genital tract (Zhou et al. 2019a; Brewster et al. 2016), ovarian, intratumoral (Wang et al. 2020; Banerjee et al. 2017; Shanmughapriya et al. 2012; Poore et al. 2020), peritoneal (Miao et al. 2020), serum (Kim et al. 2020), and fecal (Mori et al. 2019) compartments (Table 1, Fig. 1). Oncobiosis can lead to lower diversity, as in the cases of the intratumoral microbiome [Shannon index, Simpson index, and evenness index decrease (Wang et al. 2020)], the upper genital tract microbiome [Shannon index decrease, while in the Simpson index there is a borderline increase (Zhou et al. 2019a)], and the peritoneal microbiome (Miao et al. 2020). In other compartments, such as the serum, oncobiosis does not interfere with either α and β diversity. (Kim et al. 2020). [For the explanation of the diversity indices we refer the reader to (Alpha and beta diversity [http://www.metagenomics.wiki/pdf/definition/alpha-beta-diversity]; Vida et al. 2020)].

**Table 1:** The main findings of the human oncobiome studies in ovarian adenocarcinoma

Sample type and size	Method	Changes to the microbiome and other observations	Ref.
Changes to the vaginal and cervicovaginal microbiomes			
176 women with epithelial ovarian cancer, 115 healthy controls, and 69 controls with benign gynecological conditions (aged 18–87 years)	16S RNA sequencing	Cervicovaginal bacterial communities’ poor in *Lactobacillus spp.* (Type O) were more prevalent in ovarian cancer patients compared to controls. The type O community was more prevalent in BRCA (1/2) mutation carriers. Associations were stronger in younger patients (< 40 yrs. of age)	Nené et al. 2019
117 women with ovarian cancer and 171 age- and ethnicity-matched population-based control subjects	Serovar D of chlamydia elementary bodies (EB) and IgG antibodies to CHSP60-1 ELISA assay	The probability of having ovarian cancer was 90% greater in women with the highest, compared with the lowest levels of Chlamydia-EB antibodies. There was also a monotonic trend in ovarian cancer risk associated with CHSP60-1	Ness et al. 2003
Changes to the upper genital tract of women microbiome			
25 samples from the proximal fallopian tube, fimbriae, and ovary	Sequencing the V1-V2 region of the 16S gene on the Ion Torrent platform	The composition of the microbiome from healthy individuals and the ovarian cancer patients in the upper genital tract were different	Brewster et al. 2016
25 ovarian cancer tissues and 25 normal distal fallopian tube tissues	Illumina sequencing of the V3-V4 hypervariable regions of the 16S rDNA genes	Decreased diversity and species richness in ovarian cancer Clads or species upregulated in ovarian cancer: *Proteobacteria, Acinetobacter*, *Sphingomonas, Methylobacterium* spp. Clads or species downregulated in ovarian cancer: *Firmicutes*, *Candidate_division_TM7*, *Acidobacteria*, *Candidate_division_OD1, Lactococcus* spp., *Acinetobacter lwoffii, Lactococcus piscium* *Lactococcus piscium* abundance can be used for diagnostics	Zhou et al. 2019a
Changes to the ovarian microbiome			
Six women with ovarian cancer and ten women with a noncancerous ovarian condition (three patients with uterine myoma and seven patients with uterine adenomyosis)	IHC for LPS; Deep sequencing of the V3-V4 16S rDNA region	Decreasing trends in species number, Shannon Index, Simpson Index, and Evenness Index in the ovarian cancer group Clads or species upregulated in ovarian cancer: *Aquificae, Planctomycetes, Gemmata obscuriglobus*, *Halobacteroides halobius*, and *Methyloprofundus sedimenti* Clads or species downregulated in ovarian cancer: *Crenarchaeota* The relative abundance of *Anoxynatronum sibiricum* may be associated with the tumor stage. *Methanosarcina vacuolata* may be used to diagnose ovarian cancer	Wang et al. 2020
99 ovarian cancer samples (primary and recurrent), 20 matched (tissue adjacent to the tumor deemed non-cancerous by pathological analysis) samples, and 20 unmatched control samples	PathoChip, a microarray followed by probe capture and Illumina sequencing	Differential expression of viruses (Nodaviridae, Parvoviridae), Proteobacteria (*Azorhizobium*, *Escherichia*, *Firmicutes*, *Clostridium*), fungi (*Alternaria, Malassezia, Mucor, Trichosporon*), and parasites (*Acanthamoeba, Naegleria, Taenia, Trichinella*) between the cancer and matched control groups	Banerjee et al. 2017
39 tissue samples from cancerous or healthy ovaries (mean age, 55 ± 15 years; range 40 to 70 years)	Chlamydia and human papillomavirus DNA was assessed in PCR reactions	Ovarian cancer patients had a higher prevalence of Chlamydia or HPV	Shanmughapriya et al. 2012
18,116 samples across 10,481 patients and 33 types of cancer (including ovarian cancer) from the TCGA compendium of whole-genome sequencing (WGS; n = 4,831) and whole-transcriptome sequencing (RNA-seq; n = 13,285) studies	in silico approach	*Fusobacteria* (Bacteroides, Gram-negative) count in tumors was higher compared to healthy, untransformed tissues (Poore et al. 2020)	Poore et al. 2020
Changes to the peritoneal microbiome			
Peritoneal fluid from 10 ovarian cancer patients and 20 patients with benign ovarian masses (age ≥ 30)	16S RNA sequencing of the V4 region of the 16S rDNA gene	Decreased bacterial diversity in ovarian cancer	Miao et al. 2020
Changes to the serum microbiome			
166 ovarian cancer vs. 76 patients with benign ovarian tumors	Sequencing V3-V4 hypervariable regions 16S rDNA	The genus *Acinetobacter* showed high relative abundances in ovarian cancer No difference in α and β diversity Genus-level microbiome biomarkers in combination with clinical biomarkers (CA-125) can be used for diagnostic purposes	Kim et al. 2020
Changes to the gut microbiome			
A subset of 10 Lynch syndrome patients with confirmed DNA mismatch repair pathogenic mutations developing ovarian cancer (Shih Ie and Kurman 2004) vs. 8 healthy females without a family history of cancer	V4 region of the 16S rDNA was sequenced by Illumina sequencing	In the gynecological cancer group, *Bacteroides* abundance decreased and *Firmicutes*, *Actinobacteria,* and *Proteobacteria* increased. At the family level, *Lachinospiraceae*, *Bacteroideacea,* and *Rikenelacea* decreased and *Bifidobacteriacea* and *Ruminococcacea* increased	Mori et al. 2019

*IHC* immunohistochemistry

In the vaginal and cervicovaginal parts of the genital tract, *Lactobacilli* act as gatekeepers against bacterial and certain viral vaginal infections by maintaining low pH and epithelial tight junctions, as well as, producing antimicrobial substances (Łaniewski et al. 2020). Importantly, *Lactobacilli* are protective species against ovarian cancer (Xu et al. 2020). Vaginal communities that are poor in *Lactobacillus* are more prevalent in ovarian cancer patients compared to controls. *Lactobacillus* spp. poor communities are more prevalent in BRCA (1/2) mutation carriers, suggesting a role for oncobiosis in enhancing the effects of genetic mutations. Associations are stronger in younger patients (< 40 yrs. of age) (Nené et al. 2019).

In the tumor tissue, the *Proteobacteria*/*Firmicutes* ratio increases, since the abundance of *Proteobacteria* increase (Wang et al. 2020; Zhou et al. 2019a). *Fusobacteria* (*Bacteroides*) count in tumors is higher compared to healthy, untransformed tissues (Poore et al. 2020). Both *Proteobacteria* and *Fusobacteria* are Gram-negative; hence, the microbiome gains more immunogenic character. The oncobiotic peritoneal microbiome is also rich in Gram-negative bacteria (Miao et al. 2020). In contrast, the gut oncobiome is enriched in Gram positive bacteria, as *Bacteroides* abundance decreased and *Firmicutes*, *Actinobacteria,* and *Proteobacteria* increased in the gut microbiome. At the family level, *Lachinospiraceae*, *Bacteroideacea,* and *Rikenelacea* decreased and *Bifidobacteriacea*, *Ruminococcacea* increased (Mori et al. 2019).

In addition to oncobiotic transformation, genital pathogens (e.g. *Chlamydia trachomatis* or *Nesseria gonorrhea*) increase the risk for ovarian cancer (Ness et al. 2003; Shanmughapriya et al. 2012; Xu et al. 2020; Idahl et al. 2011; Trabert et al. 2019; Xie et al. 2017; Rasmussen et al. 2017; Carvalho and Carvalho 2008). A case report (Vyas 2007) showed the synchronous occurrence of Brucellosis and ovarian cancer, underlining the association between infection of the female genital tract and ovarian cancer. Of note, although *Mycobacteria* were detected in ovarian cancer specimens and were linked to pathogenesis, recent studies provided strong evidence that *Mycobacteria* stemmed from external contaminations to samples (Poore et al. 2020; Robinson et al. 2017; Chan et al. 1996). *Lactobacilli* are key species to protect against vaginal infections and *Lactobacillus* spp. poor communities increase the risk of ovarian cancer (Nené et al. 2019). Furthermore, viral infections (HPVs, CMV, EBV, or HIV) were shown to interfere with carcinogenesis in ovarian cancer (reviewed in (Łaniewski et al. 2020; Pathak et al. 2020; Levinson et al. 2018)). Besides bacteria and viruses, fungal and parasitic signatures were shown to be associated with ovarian cancer (Banerjee et al. 2017).

An ample set of data suggests that the microbiome drives inflammation and regulates immune responses to support carcinogenesis in ovarian cancer. This is highlighted by the observation that pelvic inflammatory disease is a risk factor for ovarian cancer (Łaniewski et al. 2020; Rasmussen et al. 2017, 2013; Mert et al. 2018). Infections of the genital tract are excellent drivers of local inflammation. Inflammation can drive oncogenesis through multiple pathways, involving increased oxidative stress, the resulting DNA damage, and the accumulation of mutations. Pattern recognition receptors TLR2, 4, and 5 respond to bacterial flagellin (Rutkowski et al. 2015) or LPS (Wang et al. 2020, 2014; Kashani et al. 2020; Kelly et al. 2006; Glezerman et al. 1998; Huleihel et al. 1997; Park et al. 2017; Muccioli and Benencia 2014) and have pivotal roles in driving inflammation in ovarian cancer. Allegra and colleagues alluded to an interaction between miRNAs and the microbiome (Allegra et al. 2020). Treatment of ovarian cancer cells with *Lactobacillus lactis*, a vaginal synbiont, modulates the expression of miR-21 and miR-200b, and, subsequently, TLR4 responsiveness of CAOV-4 cells (Rahbar Saadat 2019). The activation of TLR2, 4, and 5 culminate in the activation of inflammation-associated cytokine signaling pathways in ovarian cancer and the adjacent tissues, leading to the activation of NF-kappa B signaling (Zhou 2019a). Other pattern recognition receptors may be involved in ovarian cancer development (Cheng et al. 2020), but the evidence for their involvement is weak. Tumor-associated macrophages have a pivotal role in driving ovarian cancer development (Xu et al. 2019). The colonization of the peritoneum can drive metastasis formation and likely define the site of metastasis formation in the peritoneum. Furthermore, metastasis to the bowels may involve an interplay between the microbiome of the peritoneal and gastrointestinal (fecal) compartments.

## The role of bacterial metabolites in ovarian cancer

The gut microbiome has a diverse and enormous metabolic capacity due to the large number of bacterial species and the large variability in their proportions (Magnusdottir and Thiele 2017). Bacterial metabolites or components of bacteria can act locally or enter the systemic circulation of the host and exert hormone-like effects at distant sites. Such hormone-like effects were described in the pathology of breast cancer, pancreatic adenocarcinoma, colorectal cancer, gastric cancer, hepatocellular carcinoma (Miko et al. 2016; Miko et al. 2019; Kiss et al. 2020; Kuo et al. 2016; Chen et al. 2016; Shellman et al. 2017; Yoshimoto 2013; Ravnik et al. 2020; Rossi et al. 2020; Sittipo et al. 2019). In this chapter we will review those bacterial metabolites that can potentially have role in the pathogenesis of ovarian cancer. We will review the bacterial metabolism, serum/tissue levels and receptors of these metabolites in ovarian cancer and review the possible involvement of these metabolites in the pathogenesis of ovarian cancer.

It is of note that certain metabolites may have bacterial, human, or sometimes nutritional origin, these cases were identified in the respective chapters discussing the metabolite in question. When metabolomic studies are discussed it should be noted that the source of the metabolites cannot be determined (i.e. bacterial, host or nutritional).

### Lipopolysaccharides (LPS)

Lipopolysaccharides, lypoglycans, and endotoxins are components of the bacterial outer membrane in Gram-negative bacteria (Bertani and Ruiz 2018). LPS molecules have a lipid core, which facilitates membrane attachment, to which polysaccharide chains are joined. LPS essentially protects bacterial cells against external toxins, antibiotics, and bile acids. LPS is highly immunogenic and is a pathogen-associated molecular pattern (PAMP). LPS stimulates TLR4 and TLR2 receptors (Bertani and Ruiz 2018; Lu et al. 2008).

In oncobiosis accompanying ovarian cancer, the proportions of Gram-negative bacteria and, therefore, LPS quantity increase in the cancer tissue (Wang et al. 2020). LPS plays a pivotal role in driving inflammation in ovarian cancer (Wang et al. 2020, 2014; Kashani et al. 2020; Kelly et al. 2006; Glezerman et al. 1998; Huleihel et al. 1997; Park et al. 2017; Muccioli and Benencia 2014). LPS can activate cancer cells and tumor-associated macrophages (TAM). The reactivity of cancerous tissue to LPS is higher compared to normal tissues (Glezerman et al. 1998; Huleihel et al. 1997). LPS stimulation of ovarian cancer cells induces phosphatidyl-inositol-3 kinase activation, EMT, and migration marked by the overexpression of N-cadherin, Slug, Vimentin, Snail, α-SMA, TCF, MMP2, and MMP9 (Park et al. 2017). The functionality of LPS-induced inflammation is highlighted by the fact that the blockade of TLR4 reduces ovarian cancer proliferation (Kashani et al. 2020) and TLR4 activation promotes proliferation and induces drug resistance (Kelly et al. 2006). The physical presence of certain vaginal microbes, as *Lactobacillus lactis*, can modulate the responsiveness of TLR4 though modulating the expression of miR-21 and miR-200b and, hence, decrease responsiveness of CAOV-4 ovarian cancer cells to LPS (Rahbar Saadat et al. 2019).

LPS stimulation of TAMs pushes the macrophages towards the M1 profile (Trenti et al. 2018; Wanderley et al. 2018), which is cytotoxic and cytostatic for ovarian cancer cells (Han et al. 1999). The applicability of LPS stimulation to induce and immunogenic destruction of ovarian cancer cells was questioned by recent results showing that, in an experimental model of ovarian cancer, LPS administration did not prolong survival, and, based on the timing of administration, may have even shortened survival (Vindevogel et al. 2016). Taken together, LPS appears to be a procarcinogenic bacterial metabolite.

### Lysophospholipids

Lysophospholipids are by-products of metabolic reactions involved in bacterial membrane homeostasis (Zhang and Rock 2008; Zheng et al. 2017), as well as, are synthesized in the cells of the host. Gram-negative bacteria have high lysophospholipid content (Zhang and Rock 2008; Zheng et al. 2017) and, as stated earlier, the proportions of Gram-negative bacteria increase in ovarian cancer patients (Wang et al. 2020). Their chemical structure differs from general phospholipids. The cone-shaped structure of lysophospholipids confers detergent-like properties to these molecules (Zheng et al. 2017). Lysophospholipids are generated under stress conditions, either by phospholipase A2, which removes a fatty acid moiety from position 2 of glycerol, as by-products of phospholipid biosynthesis, or by the release of exogenous lipases (Zheng et al. 2017). Lysophosphatids bind to lysophosphatidic acid receptors (LPAR1-6) (Lin et al. 2010). Lysophosphatids are present in the serum, plasma, and ascites (Ye et al. 2008).

Lysophosphatids impact the behavior of ovarian cancer cells by influencing multiple cancer hallmarks. Lysophosphatidic acid (LPA) and lysophosphatidylserine induce Akt, MAPK, and calcium signaling and LPA induces cell proliferation, migration, and invasion of ovarian cancer cells (Xu et al. 1995; Estrella et al. 2007; Jeong et al. 2012, 2013; Pustilnik 1999; Sengupta et al. 2003; Hurst and Hooks 2009). LPA can upregulate the expression of elements of angiogenesis in ovarian cancer (Lee et al. 2006). Lysophosphatids are upregulated in the plasma of ovarian cancer patients (Fanet al. 2012; Zhang et al. 2013). TLR5 activation enhances the formation of distal metastases in ovarian cancer by reprograming the immune system (Rutkowski et al. 2015). Lysphosphatids are carcinogenic metabolites similar to LPS.

### Tryptophan metabolites

The metabolism of tryptophan, an amino acid, is very complex and intricate. Approximately 4–6% of tryptophan undergoes bacterial metabolism and yields indole-derivatives (Wikoff et al. 2009; Yokoyama and Carlson 1979; Browne et al. 2012; Aidy et al. 2012; Mardinoglu et al. 2015; Gao 2018). Bacterial tryptophan metabolism has multiple arms, as reviewed in (Wikoff et al. 2009; Yokoyama and Carlson 1979; Browne et al. 2012; Aidy et al. 2012; Mardinoglu et al. 2015; Gao et al. 2018). The main receptors for tryptophan-derivatives are aryl hydrocarbon receptor (AHR) and pregnane X receptor (PXR) (Zelante et al. 2013; Venkatesh et al. 2014; Lamaset al. 2016).

AHR has pivotal roles in immune regulation (Gao et al. 2018; Kim et al. 2018) and low dietary tryptophan leads to immunosuppression (Sonner et al. 2019). Mucosal immunity can be regulated by AHR activation. Therefore, indole-derivatives can impact on the composition of microbiome compartments. As an example, indol-derivatives support the growth of *Lactobacillus reuteri* that, in turn, inhibit the expansion of pathogenic bacteria (Zelante et al. 2013; Shi et al. 2007; Qiu et al. 2012; Zhang et al. 2017) and protect against ovarian cancer (Nené et al. 2019). In addition, certain *Lactobacilli* can utilize tryptophan as an energy source. Therefore, a tryptophan-rich diet can improve *Lactobacillus* viability and induce proliferation (Zelante et al. 2013).

Tryptophan levels and indolepropionic acid (a bacterial tryptophan metabolite) decrease in the serum of ovarian cancer patients (Plewa et al. 2017; Hilvo et al. 2016; Zhou et al. 2010; Zhang et al. 2012; Ke et al. 2015), a trend that is aggravated by increased stage of the disease (Ke et al. 2015). In good agreement with that, urinary indolepropionic acid concentrations correlate with the presence of epithelial ovarian cancer compared to healthy controls (Zhang et al. 2013). Apparently, indole-derivatives are antineoplastic and their production decreases in ovarian cancer.

### Other bacterial metabolites with potential involvement in ovarian cancer

Other bacterial metabolites were shown to affect other cancers, nevertheless, based on the currently available data, their involvement in ovarian cancer was ambiguous. For metabolism and species information we refer the readers to reviews (Miko et al. 2019; Kiss et al. 2020; Wortham et al. 2007; Michael et al. 2018; Ridlon et al. 2006; Ridlon and Bajaj 2015; Gerard 2013; Ramirez-Perez et al. 2017).

*Short chain fatty acids (SCFAs)*, encompassing acetate, propionate, butyrate, and lactate were cytostatic in numerous cancers. The reference concentration of SCFAs in the human serum is in the range of 10–100 µM (Clausen et al. 1991; Jakobsdottir et al. 2013; Ktsoyan et al. 2016) and may reach locally to 1 mM (Pryde et al. 2002). SCFAs act on free fatty acid receptors (FFARs) and AHR (Jin et al. 2017). Most SCFAs can act as energy sources in cells (Sittipo et al. 2019) or may inhibit histone deacetylases and through that, can modulate epigenetics (Shimazu 2013; Menzies et al. 2016; Fellows and Varga-Weisz 2020). SCFAs impact on the pH of the colon, modulate the immune system and, as a consequence, influence the composition of the colon microbiome (Fachi et al. 2020). SCFA production probably plays role in quorum sensing, as suggested by in vitro experiments (Li et al. 2020; Ge et al. 2019). When ovarian cancer cells were treated with SCFAs in superphysiological, low millimolar concentrations (1–5 mM) in in vitro experiments, SCFAs exerted cytostatic pro-apoptotic (Terao et al. 2001; Krupitza et al. 1995), anti-EMT (Mrkvicova 2019) features and inhibited invasiveness (Krupitza et al. 1996) and induced senescence (Terao 2001; Yabushita and Sartorelli 1993; Langdon et al. 1988). These results suggest that SCFAs can potentially be antineoplastic. Contradicting these beneficial effects, metabolomic studies showed that hydroxybutyric acid (Hilvo et al. 2016), lactate and pyruvate (Kyriakides et al. 2016; Boss et al. 2000; Fong et al. 2011) increased in tumors and cyst fluid of patients. The bacterial pathways generating SCFAs (pentose phosphate pathway, starch and sucrose metabolism, fructose and mannose, pyruvate metabolism, galactose metabolism, and glycan degradation) (Wang et al. 2020) are upregulated in the tumors of ovarian cancer patients, as well as, in the tumor cells themselves (Turkoglu et al. 2016).

*Polyamines* (e.g. spermine, spermidine) are organic molecules with more than two amine groups. Polyamines support bacterial growth and biofilm formation and through these, in pathogenic species, polyamines are virulence factors (Michael et al. 2018) and quorum sensing signals (Rattanaphan et al. 2020; Inaba et al. 2020). Some circulating polyamines may be of bacterial, human, and/or dietary origin (Ramos-Molina et al. 2019). Polyamines and putrescine are usually linked with tumorigenesis in cancers other than ovarian cancer. A serum metabolome study demonstrated that polyamine metabolism was dysregulated in ovarian cancer (Zhou 2010). Spermine and spermidine levels in erythrocytes, plasma, and urine of ovarian cancer patients increase compared to healthy, age-matched controls, suggesting a systemic increase in these metabolites in ovarian cancer (Hayase et al. 1985; Lawton et al. 1989; Chanda and Ganguly 1995). Similarly, urinary *N*^1^,*N*^12^-diacetylspermine levels are higher in patients with malignant ovarian tumors compared to patients with benign tumors (Niemi et al. 2017). Furthermore, the probability/frequency of increased blood polyamine levels is in line with the progression of ovarian cancer.

*Secondary bile acids* (lithocholic acid (LCA), deoxycholic acid (DCA), and ursodeoxycholic acid (UDCA)) are bacterial transformation products of hepatic primary bile acids taking place in the intestines. Bile acids undergo enterohepatic circulation (hepatic synthesis and secretion to duodenum, intestinal transformation, reabsorption and return to the liver) that is hampered in ovarian cancer. The enterohepatic circulation of bile acids is modified in ovarian cancer; bile acid reabsorption is hampered (Larsen et al. 2019) and less bile acids appear in the ascites (Hedenborg et al. 1988). A fraction of the reabsorbed bile acids enter the systemic circulation (Marshall et al. 2016) (total bile acid concentration in the serum is > 5 µM in a healthy individual) and that bile acid pool can exert hormone-like, systemic effects (Miko et al. 2019, 2018; Ravnik et al. 2020; Watanabe et al. 2006; MahmoudianDehkordi 2019; Sarin et al. 2019; Tang et al. 2019). The serum concentration of bile acids is submicromolar for deoxycholic acid and 100–300 nM for ursodeoxycholic acid, while LCA is present in much lower concentrations in serum, ~ 30 nM (Miko et al. 2018). Bile acids can impact on the composition of the microbiome (Tsuei et al. 2014; Merritt and Donaldson 2009; Garcia-Quintanilla et al. 2006; Prieto et al. 2006, 2004; Kandell and Bernstein 1991; Schaffler and Breitruck 2018; Sorg and Sonenshein 2010) and bile acids can facilitate bacterial translocation into tissues (Slocum et al. 1992). Bile acid signaling in humans is very complex, with multiple receptors. Receptors for bile acids include farnesyl-X-receptor (FXR), liver-X receptor (LXR), *Takeda* G Protein-Coupled Receptor 5 (TGR5), constitutive androstane receptor (CAR), vitamin D receptor (VDR), pregnane X receptor (PXR), sphingosine-1-phosphate receptor 2 (S1PR2), and muscarinic M2,3 receptors. Apart from TGR5, S1PR2, M2, and M3, all receptors are nuclear receptors.

Most in vitro cellular studies assessed bile acids at superphysiological concentrations (0.05–400 mM) (Horowitz et al. 2007; Schuldes et al. 2001; Jin et al. 2018). At these superphysiological concentrations, bile acids are cytotoxic, antineoplastic in cell models (Horowitz et al. 2007; Schuldes et al. 2001), mostly due to changing the biophysical properties of the cell membrane and damage to DNA (Schuldes et al. 2001), although, whether these changes would occur at physiological concentrations require further studies. The concentrations of most bile acids as 3b-hydroxy-5-cholenoic acid, glycoursodeoxycholic acid, and deoxycholic acid (Ke 2015), taurochenodeoxycholic acid (Fan et al. 2016) decrease in ovarian cancer patients. Bile acid receptors have variable effects on ovarian cancer cells. The activation of a bile acid receptor LXR reduces the proliferation of ovarian carcinoma cells (Scoles et al. 2010; Rough et al. 2010) and improves the efficacy of anti-VEGF therapy (Curtarello et al. 2019). In contrast to that, the inhibition of the PXR pathway induces ovarian cancer cell proliferation (Masuyama et al. 2016). Furthermore, PXR or CAR activation contributes to chemoresistance and proliferation (Wang et al. 2014; Gupta et al. 2008; Chen et al. 2012). Taken together, these results suggest that bile acids may have cytostatic properties on ovarian cancer cells, and this effect is lost or reduced in ovarian cancer patients. However, the role of the bile acid receptors calls for further detailed investigations.

## Modulating the oncobiome in ovarian cancer

### Antibiotics

Topical or systemic antibiotic treatment has a strong impact on the composition of the microbiome. Antibiotic treatment impacts the frequency and recurrence of breast cancer (Friedman et al. 2006; Kirkup et al. 2019, 2020; Wirtz et al. 2013). In the case of pancreatic adenocarcinoma where bacterial colonization of the pancreas is a major driver of carcinogenesis (Kiss et al. 2020) similar to ovarian cancer, antibiotic treatment was beneficial in animal models (Thomas et al. 2018). Pathogen colonization influences ovarian carcinogenesis, indicating that antibiotic use in ovarian cancer may inhibit cancer cell movement and metastasis formation. However, we are not aware of any dedicated in vivo study to assess this possibility, nevertheless, the literature suggests that antibiotic treatment may impact on the gut microbiome and, hence modulate the polarization of the immune system, and through that inflammation promoting ovarian cancer (Cheng et al. 2020).

Available studies used antibiotics and antimycotics as single agents or in drug combinations to directly act on cancer cells in in vitro models to improve chemotherapy. Lamb and colleagues (Lamb et al. 2015) showed that a set of antibiotics (erythromycins, tetracyclines, glycylcyclines, and chloramphenicol) can block cell proliferation and reduce the proportions of ovarian cancer stem cells. Minocycline, as a single agent, can also reduce the proliferation of ovarian cancer cells by interfering with energy-sensing pathways and proliferative signaling (Ataie-Kachoie et al. 2013a, b, 2015; Pourgholami et al. 2013). Ciprofloxacin can also act as a single agent to reduce cancer cell proliferation (Kloskowski et al. 2010). In addition, ciprofloxacin prophylaxis in taxol-based chemotherapy regimens prevents febrile neutropenia and sepsis during chemotherapy (Carlson et al. 1994). Finally, salinomycin can impair cancer cell proliferation by inhibiting proliferation, inducing apoptosis, blocking EMT, and reducing stem-ness (Zhang et al. 2012; Parajuli et al. 2013a, b; Chung et al. 2016; Kaplan and Teksen 2016; Li et al.^2017^; Lee et al. 2017). Several of these antibiotics can bind to mitochondrial complex I and interfere with cellular energetics (Lamb et al. 2015) to interfere with the behavior of cancer cells.

Antibiotics can be used as a component of drug combinations also. Tigecycline (Tan et al. 2017), clarithromycin (Zhou et al. 2019b), amphotericin B (Kojima et al. 1994), valinomycin (Daoud and Forde 1991), and salinomycin (Michalak et al. 2020) can be used in combination with cisplatin. Moreover, antibiotics can be used to counteract cisplatin resistance, a major issue in ovarian cancer chemotherapy, as evidenced in murine models (Chambers et al. 2020). Minocycline can potentiate topoisomerase inhibition (Huang et al. 2018).

Interestingly, a study by Wang et al. (Wang et al. 2020) showed that the resident bacteria in ovarian cancer tissue produced antibiotics. In particular, the biosynthesis of butirosin, neomycin, vancomycin, streptomycin, and ansamycins were different in cancerous tissues compared with healthy control tissues. The biological impact of this finding has not been assessed to date.

Although many studies suggest the potential applicability of antibiotics in cancer therapy, Xu and colleagues (Xu et al. 2019) showed that combination treatment of Balb/c mice grafted with SKOV4 ovarian cancer cells with ampicillin, vancomycin, neomycin, and metronidazole promoted the growth and invasiveness of grafts. Thus, the applicability of antibiotics should be carefully assessed and considered in a clinical setting. In addition, the applicability of oral or vaginal probiotics should be considered (Brewster et al. 2016; Chase et al. 2015; Champer et al. 2018), although experimental data is missing.

### Nutrients and diet

Nutrition modulates the composition of the microbiome along with other lifestyle elements. Obesity is a risk factor for ovarian cancer (Leitzmann et al. 2009) and the ketogenic diet was shown to reduce central obesity and reduce insulin levels in ovarian cancer patients (Cohen et al. 2018). Animal fat (Shu et al. 1989) and retinol (Zhang et al. 2004) consumption increases the risk for ovarian cancer (Shu et al. 1989; Zhang et al. 2004), while vegetable (Shu et al. 1989), fiber (Zhang et al. 2004), carotene (Zhang et al. 2004), vitamin C (Zhang et al. 2004), and vitamin E (Zhang et al. 2004) consumption is protective in a dose-dependent fashion. Nutrients (e.g. polyamines (Ramos-Molina et al. 2019; Tofalo et al. 2019) or tryptophan (Gao et al. 2018; Lin et al. 2017)) can directly impact inflammation, a driver of carcinogenesis in ovarian cancer (Madeo et al. 2020). Polyunsaturated fatty acids reduce the expression of key chemokines (e.g. IL-6) in ovarian cancer-associated. *Lactobacilli* seem to be unique in the human vaginal flora (Miller et al. 2016) and are protective against ovarian cancer (Nené et al. 2019). In good agreement with this, animal-derived nutrients increase the risk of cervical cancer by reducing *Lactobacilli* in the vaginal flora (Seo et al. 2016) and tryptophan supplementation that support the growth of certain *Lactobacilli* by serving as an energy source can improve *Lactobacillus* viability and induce proliferation (Zelante et al. 2013).

### Interference with chemotherapy

The microbiome can interfere with cancer chemotherapy and management strategies. In fact, the interactions form a circuit, where (1) the microbiome or oncobiome interferes with the metabolism of chemotherapeutic drugs, modulates the immune system, and interferes with the side effects of drugs, and, conversely, (2) therapy modulates the composition and behavior of the microbiome. We will review the interference between the microbiome and the individual elements of the chemotherapy regimen used in ovarian cancer. Bacterial metabolites themselves can modulate the effectiveness of chemotherapeutic agents that is summarized in Table 2.

**Table 2:** Interactions between bacterial metabolites and drugs relevant in ovarian cancer chemotherapy

Drug	Metabolite	Effect	Ref.
Cisplatin	Spermine, spermidine	induce cisplatin resistance	Marverti et al. 2005; Marverti et al. 2004; Marverti et al. 2001; Marverti et al. 1997; Hector et al. 2004; Desiderio et al. 1997
	Butyrate and valproic acid		Mrkvicova et al. 2019; Wasserman et al. 1989; Sajadpoor et al. 2018
Paclitaxel	LPS	TLR4 activation induces paclitaxel chemoresistance	Kelly et al. 2006; Edwardson et al. 2017; Huang et al. 2014; Szajnik et al. 2009
Doxorubicin/Adriamycin	Taurochenodeoxycholate	sensitizes resistant cells	Schuldes et al. 2001
	Butyrate and valproic acid		Wasserman et al. 1989
	Spermine	induces Doxorubicin resistance	Schuldes et al. 2001
Niraparib	Butyrate and valproic acid	sensitizes resistant cells	Booth et al. 2018
Topoisomerase II inhibitors (TopoIIi)	Spermine, spermidine	sensitizes cells to TopoIIi	Desiderio et al. 1997
Mitomycin	Taurochenodeoxycholate	sensitizes	Schuldes et al. 2001

*TopoIIi* -Topoisomerase II inhibitor

To date, there is no data on the microbial metabolism of medically used *taxols*. Paclitaxel can bind to and activate TLR4 receptors to reprogram the immune system (Byrd et al. 1999). This may be the reason why the colonization of the tumor with *Salmonella typhimurium* or *Porphyromonas gingivalis* (Miyake et al. 2019; Woo et al. 2017) can interfere with paclitaxel efficiency in cancers other than ovarian cancer. In the same manner, bacterial LPS may confer resistance to Taxol in macrophages (Sweet and Hume 1996), hence, colonization of ovarian cancer tissue by LPS-rich, Gram-negative bacteria can impact on local Taxol effectiveness. Taxanes can interfere with the invasiveness and infectivity of *Klebsiella pneumoniae* (Oelschlaeger and Tall 1997) and *Campylobacter jejuni* (Biswas et al. 2000).

Platinum drugs can crosslink (Dedduwa-Mudalige and Chow 2015) and do oxidative damage to nucleic acids in bacterial, or in human cells (Beaufay et al. 2020). Cisplatin and carboplatin, therefore, exert bacteriostatic properties on *Acinetobacter*, *Mycobacteria*, and *Pseudomonas aeruginosa* (Yuan et al. 2018; Gajdács and Spengler 2019; McCarron et al. 2012; Zhang et al. 2011) and other pathogens (Hummell and Kirienko 2020). Cisplatin kidney toxicity can be prevented via limiting uremic toxins production by probiotics, such as *Lactobacillus salivarius* BP121 (Lee et al. 2020), a mixture of *Lactobacillus plantarum plantarum*, *Lactobacillus paracasei paracasei*, and *Streptococcus salivarius, or Streptococcus thermophilus* (Lee et al. 2020). Cisplatin administration compromises epithelial barriers, leading to bacterial translocation (Perales-Puchalt et al.^2018^). Cisplatin resistance can be alleviated by co-treatment with antibiotics (Tan et al. 2017; Zhou et al. 2019b; Kojima et al. 1994; Daoud and Forde 1991; Michalak et al. 2020; Chambers et al. 2020).

Bacteria can metabolize TopoII inhibitors through β-glucuronidase enzymes (Roberts et al. 2013; Wallace et al. 2015; Bhatt et al. 2020), a key factor influencing TopoII inhibitor availability (Bhatt et al. 2020) and toxicity (Roberts et al. 2013). In fact, β-glucuronidase enzymes can deconjugate and reactive estrogens and, hence, increase estrogen recirculation (Flores et al. 2012; Baker et al. 2017; Ervin et al. 2019) suggesting a link between bacterial estrogen recycling and TopoII inhibitor availability. TopoII inhibitors have bacteriostatic properties (Patel et al. 1998) and, not surprisingly, TopoII inhibitors modulate the gut microbiome. Irinotecan treatment in rats increased the abundance of clostridial clusters I and XI and *Enterobacteriaceae*, while total bacteria, *Clostridium* cluster VI, and the *Bacteroides*-group decreased. These effects were prevented by oral glutamine administration (Lin et al. 2012). TopoII inhibitors interfere with TLR4 (Wardill et al. 2016) and SCFA (Irinotecan 2006; Encarnação et al. 2018; Lin 2014) signaling. The microbiome plays a key role in mediating the severity of TopoII inhibitor-induced mucositis (Ribeiro et al.^2016^; Pedroso et al. 2015; Wang et al.^2019^), which can be ameliorated by *Escherichia coli* Nissle 1917 (a probiotic) (Wang et al. 2019) or butyrate (Encarnação et al. 2018) and its prebiotics (Lin et al. 2014). The involvement of butyrate demonstrates the contribution of SFCA signaling.

Anthracyclines (e.g. Doxorubicin) are biosynthesized by *Streptomyces* strains and are used as intercalating agents in cytostatic therapy. Anthracyclines can act as antibiotics too (Cox et al. 2014), for example, anthracyclines can inhibit the growth of *Acinetobacter* species (McCarron et al. 2012). Multiple bacterial species can metabolize and inactivate anthracyclines (Parajuli et al. 2018; Dhakal et al. 2018; Zabala et al. 2013). Furthermore, anthracycline treatment can facilitate the loss of the intestinal barrier, bacterial translocation and bacterial entry to secondary lymphoid organs (Alexander 2017).

The silencing of PARP1 increases the diversity of the gut microbiome (Vida et al. 2018; Larmonier et al. 2016), indicating that PARP inhibitors may also increase microbiome diversity. PARP enzymes play a role in TLR4 and 5 (Liaudet et al.^2002^; Zerfaoui et al. 2010), AHR (Diani-Moore et al. 2010; Macpherson et al. 2013), and SCFAs signaling through AHR (Jin et al. 2017).

Bevacizumab is a monoclonal antibody targeting VEGF to inhibit vascularization. Bevacizumab is the only tool in targeted therapy to be applied in ovarian cancer. Nevertheless, there are novel, experimental immunological approaches in the treatment of ovarian cancer adoptive cell transfer (ACT) (Rosenberg et al. 2008; Kershaw et al. 2006), Chimeric antigen receptor (*CAR*) *T*-cell therapy (CAR-T) (Schepisi 2021), dendritic cell vaccines (Zhang et al. 2020), immune-checkpoint blockade that aim to enhance T cell responses (Schepisi 2021). The effectiveness of therapies involving the activation of the immune system depend on the composition and immunogenic properties of the microbiome (Routy et al. 2018a; Vetizou et al. 2015; Sivan et al. 2015; Matson et al. 2018; Innao et al. 2020; Brandi and Frega 2019; Sun et al. 2020). It should be noted that ovarian cancer-specific microbiome data is missing.

## Conclusions

**Fig. 2:**
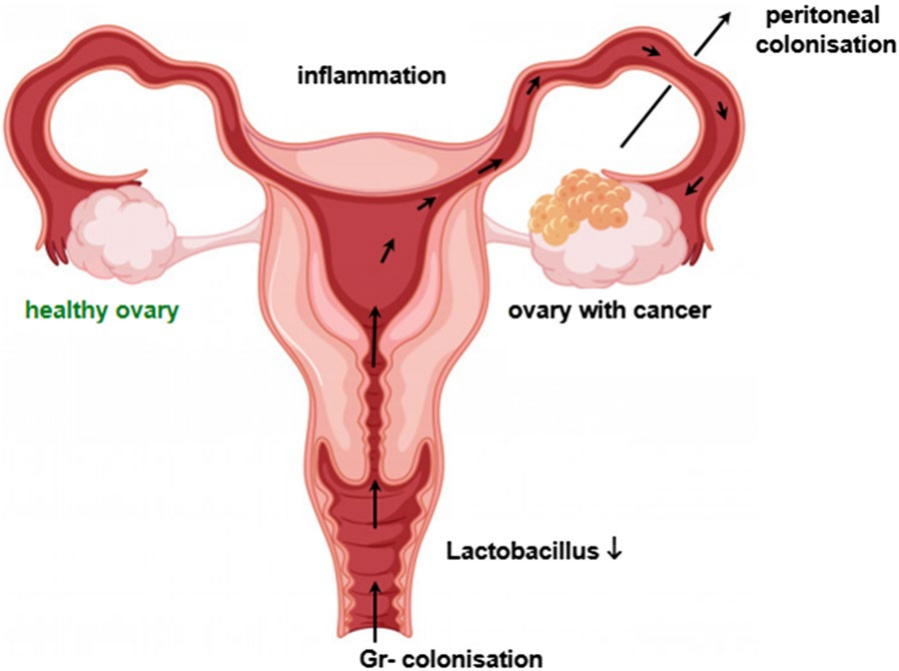
Bacterial colonization of the upper genital tract as a risk factor for ovarian cancer. In black, the microbial risk factors of ovarian cancer. The image is a free-use image from https://image.freepik.com/free-vector/woman-ovarian-cancer-concept-drawing_1308-15806.jpg

Current data support the oncobiosis of multiple microbiome compartments in ovarian cancer. Vaginal infections and the colonization of the upper genital tract seem to play important roles in the development of ovarian cancer (Fig. 2) primarily by supporting tumor-promoting inflammation. We provided evidence that signaling through bacterial metabolites play a role in the pathogenesis of ovarian cancer, a set of proinflammatory metabolites (LPS, lysophosphatides) are upregulated, while tryptophan metabolites were downregulated that have antineoplastic features. It should be noted that further studies are needed to define the involvement of metabolite signaling in ovarian cancer.

The association of oncobiosis with ovarian cancer implies the possible use antibiotics or probiotics to mitigate the side effects of chemotherapy (Wang et al. 2019) or eradicate the colonizing bacterial species. Along the same lines, supplementing chemotherapy in hyperthermic intraperitoneal chemotherapy should be considered. To our surprise, such studies are largely missing, despite the fact that the literature discusses numerous hypotheses (Brewster et al. 2016). Probiotics are frequently used to treat banal vaginal infections. Therefore, conducting studies of these agents in relation to ovarian cancer would be straight forward, similar to assessing the use of antibiotics in ovarian cancer.

Another field, where bacterial dysbiosis and metabolite signaling can be exploited, is therapy and diagnostics. There are numerous studies in the literature indicating that microbiome profiles in the intratumoral area (Wang et al. 2020; Poore et al. 2020), genital tract (Wang et al. 2020; Zhou et al. 2019a), the serum (Kim et al. 2020), or the peritoneum (Miao 2020) can be exploited as biomarkers to diagnose ovarian cancer. Metabolomics studies also revealed exploitable biomarkers (Turkoglu et al. 2016). Beyond early diagnostics, these biomarkers can be used for screening, prognosis, patient stratification (e.g. for drug effectiveness), and prognosis. The practical applicability of bacterial metabolite signaling in view of our current understanding of bacterial metabolite signaling warrant future studies.

## Search strategy and selection criteria

References to this review were identified through the prior knowledge of the authors that was complemented by systematic search of Pubmed by using the combinations “microbiome—ovarian cancer”, “ovarian cancer—metabolomics”. Species information, bacterial metabolism was described based on the prior knowledge of the authors and were updated through Pubmed search. Pubmed search was performed with the name of bacterial metabolites + ovarian cancer, name of the metabolite receptor + ovarian cancer. Articles published in English were included with no restriction on publication date.

## Data Availability

Not applicable.
